# Supervised training of laparoscopic colorectal cancer resections does not adversely affect short- and long-term outcomes: a Propensity-score-matched cohort study

**DOI:** 10.1186/s12957-022-02560-y

**Published:** 2022-03-29

**Authors:** Manfred Odermatt, Jim Khan, Amjad Parvaiz

**Affiliations:** 1 Stadtspital Zuerich Triemli (City Hospital Zurich Triemli), Birmensdorferstrasse 497, 8063 Zurich, Switzerland; 2 Portsmouth Hospitals University NHS Trust, Southwick Hill Road, Cosham, Portsmouth, Hampshire, PO6 3LY UK; 3 Poole Hospital NHS Foundation Trust, Longfleet Road, Poole, Dorset, BH15 2JB UK

**Keywords:** Colorectal, Laparoscopy, Supervised training

## Abstract

**Background:**

Supervised training of laparoscopic colorectal cancer surgery to fellows and consultants (trainees) may raise doubts regarding safety and oncological adequacy. This study investigated these concerns by comparing the short- and long-term outcomes of matched supervised training cases to cases performed by the trainer himself.

**Methods:**

A prospective database was analysed retrospectively. All elective laparoscopic colorectal cancer resections in curative intent of adult patients (≥ 18 years) which were performed (non-training cases) or supervised to trainees (training cases) by a single laparoscopic expert surgeon (trainer) were identified. All trainees were specialist surgeons in training for laparoscopic colorectal surgery. Supervised training was standardised. Training cases were 1:1 propensity-score matched to non-training cases using age, American Society of Anesthesiologists (ASA) grade, tumour site (rectum, left and right colon) and American Joint Committee on Cancer (AJCC) tumour stage. The resulting groups were analysed for both short- (operative, oncological, complications) and long-term (time to recurrence, overall and disease-free survival) outcomes.

**Results:**

From 10/2006 to 2/2016, a total of 675 resections met the inclusion criteria, of which 95 were training cases. These resections were matched to 95 non-training cases. None of the matched covariates exhibited an imbalance greater than 0.25 (│*d*│>0.25). There were no significant differences in short- (length of procedure, conversion rate, blood loss, postoperative complications, R0 resections, lymph node harvest) and long-term outcomes. When comparing training cases to non-training cases, 5-year overall and disease-free survival rates were 71.6% (62.4–82.2) versus 81.9% (74.2–90.4) and 70.0% (60.8–80.6) versus 73.6% (64.9–83.3), respectively (not significant). The corresponding hazard ratios (95% confidence intervals, *p*) were 0.57 (0.32–1.02, *p* = 0.057) and 0.87 (0.51–1.48, *p* = 0.61), respectively (univariate Cox proportional hazard model).

**Conclusions:**

Standardised supervised training of laparoscopic colorectal cancer procedures to specialist surgeons may not adversely impact short- and long-term outcomes. This result may also apply to newer surgical techniques as long as standardised teaching methods are followed.

## Background

In recent years, teaching laparoscopic colorectal procedures to colorectal consultants only proficient in open surgery has been undertaken to popularise the minimally invasive method and make it available for a broader range of patients [1, 2]. The short-term benefits of laparoscopy on recovery, pain and length of hospital stay whilst not affecting oncological outcomes have made the method equally attractive for healthcare providers and patients [3, 4]. Increasingly, novice surgeons are now primarily trained in laparoscopy, and because many of them do not have extensive prior experience in open surgery, the teaching approach is generally more stepwise and includes simulation training as well [5]. Because colorectal consultants are supposed to have more surgical experience, a steep learning curve is expected from them to adopt the method in a timely manner due to the fact that training sessions in theatre under guidance and interaction by a proficient laparoscopic surgeon (supervised training) is considered to be a time-consuming and therefore costly undertaking [6]. Concern may arise that those training situations may have put the patient at an increased risk for intraoperative and postoperative complications as well as for a worse oncological resection quality, potentially resulting in earlier relapse or death in the long term [7]. Hence, some laparoscopic colorectal surgeons may not offer systematic training to colleagues in the hope of avoiding additional risks or increasing their own case numbers. A positive association of the surgeon’s case load and improved short-term outcomes in colonic cancer surgery has been demonstrated [8, 9]. Reflecting a 10-year period of formal supervised training of consultants and fellows in laparoscopic colorectal resections, we wanted to determine whether colorectal cancer patients used for training purposes (training cases) were adversely affected in the short and, in particular, the long term when compared to matched patients with similar risk profiles and procedures primarily operated on by the laparoscopic trainer himself (non-training cases). We hypothesised that training cases and non-training cases have the same postoperative outcomes

## Methods

### Study design, setting and participants

This is a retrospective analysis of a prospectively collected database of a patient cohort who underwent colorectal cancer resections performed by a single laparoscopic expert surgeon himself (non-training cases) or provided as training operations (training cases) to consultants or fellows (trainees) supervised by the same laparoscopic expert surgeon (trainer) in the Queen Alexandra Hospital, Portsmouth, UK, from September 2006 to February 2016. The Queen Alexandra Hospital is a community-based healthcare provider where 200–300 colorectal cancer resections per year are performed. It is a national training centre for colorectal laparoscopic surgery, and the surgeon performing (non-training cases) or supervising (training cases) the investigated procedures is an accredited trainer of a National Training Programme for Laparoscopic Surgery (Lapco) [10] which had the aim back in 2008 to increase the number of laparoscopically trained colorectal surgeons in England according to the National Institute of Health and Clinical Excellence (NICE) recommending laparoscopic resection as an alternative to open resection [11].

Inclusion criteria for this study were adult (≥ 18 years of age) colorectal cancer patients who were operated on by elective conventional laparoscopy in curative intent by the trainer himself (non-training cases) or by trainees supervised by the same trainer (training cases) during the study period. The included cases were grouped into training cases and non-training cases. The training cases supervised by the mentioned surgeon were 1:1 propensity-score matched to non-training cases for age, American Society of Anesthetists grade (ASA), site of tumour (rectum, left colon or right colon) and American Joint Committee on Cancer (AJCC) stage as covariates. The matching was performed applying the nearest-neighbour method without discards and no calliper and aimed to achieve standardised mean differences of covariates that did not exhibit 0.25 between the two groups. After cancer resection, patients were enrolled in a regular follow-up every 6 months for 2 years, followed by yearly appointments for at least a further 3 years. A colonoscopy was scheduled 1 and 4 years after surgery, and a computed tomography scan was performed on a yearly basis. Further investigations were initiated at any time when symptoms of potential recurrence occurred. Operative, postoperative in-hospital and follow-up data were collected in a structured database by a full-time research assistant who also was responsible for data validation and regular checks of death dates using official registries.

### Variables, data sources, bias, study size and quantitative variables

Primary outcomes were overall and disease-free survival. Secondary outcomes were operative parameters such as conversion to open surgery, blood loss and length of procedure; postoperative complications graded according to Clavien–Dindo [12], length of stay and readmission; and oncological surrogate parameters such as status of resection margins (R stage) and lymph node harvest. Although being of value in predicting early anastomotic leak, procalcitonin was not determined routinely [13]. Data sources were original documents like patient notes, reports of investigations (endoscopy, radiology, pathology, laboratory), treatment reports (surgery, oncology, radiotherapy) and official registries to check death dates. Due to being potential confounders, baseline patient characteristics (Table 1 for detailed parameter list), including oncological and procedural details, were analysed in detail in order to be able to perform sensitivity analyses in case of suspected bias. The study size finally resulted from identified training cases and the matched non-training cases that met the inclusion criteria. None of the quantitative variables was categorised.

**Table 1:** Baseline characteristics before and after 1:1 propensity-score matching of training case to non-training case colorectal cancer resections

	Non-training cases before matching (*n* = 580)	Non-training cases after matching (*n* = 95)	Training cases (*n* = 95)	*p*
Age (median, range) in years	70 (29–92)	72 (29–90)	71 (27–92)	0.677^a^
Gender				0.191^b^
Male	320 (55.2%)	55 (57.9%)	46 (48.4%)	
Female	260 (44.5%)	40 (42.1%)	49 (51.6%)	
BMI^1^ (kg/m^2^)	26 (17–52)	26 (17–43)	26 (17–47)	0.959^a^
ASA^2^				0.653^b^
1	58 (10.0%)	9 (9.5%)	13 (13.7%)	
2	379 (65.3%)	66 (69.5%)	62 (65.3%)	
3	141 (24.3%)	20 (21.1%)	20 (21.1%)	
Tumour area				0.821^b^
Rectum	223 (38.4%)	28 (29.5%)	32 (33.7%)	
Left-sided colon	172 (29.7%)	28 (29.5%)	26 (27.4%)	
Right-sided colon	185 (31.9%)	39 (41.1%)	37 (38.9%)	
Previous surgery				0.424^b^
No	391 (67.4%)	65 (68.4%)	70 (73.7%)	
Yes	189 (32.6%)	30 (31.6%)	25 (26.3%)	
T stage				0.250^c^
T0	6 (1.0%)	2 (2.1%)	0 (0.0%)	
T1	58 (10.0%)	8 (8.4%)	9 (9.5%)	
T2	134 (23.1%)	18 (18.9%)	22 (23.2%)	
T3	323 (55.7%)	54 (56.8%)	59 (62.1%)	
T4a	51 (8.8%)	12 (12.6%)	5 (5.3%)	
T4b	8 (1.4%)	1 (1.1%)	0 (0.0%)	
N stage				0.346^c^
N0	366 (63.1%)	57 (60.0%)	65 (68.4%)	
N1	140 (24.1%)	24 (25.3%)	22 (23.2%)	
N2	74 (12.8%)	14 (14.7%)	8 (8.4%)	
AJCC^3^ tumour stage				0.523^c^
0	4 (7%)	1 (1.1%)	0 (0.0%)	
1	150 (25.9%)	20 (21.1%)	23 (24.2%)	
2	201 (34.7%)	35 (36.8%)	40 (42.1%)	
3	203 (35.0%)	36 (37.9%)	27 (28.4%)	
4	22 (3.8%)	3 (3.2%)	5 (5.3%)	
Radiotherapy				0.771^c^
None	534 (92.1%)	88 (92.6%)	87 (91.6%)	
Short course	13 (2.2%)	2 (2.1%)	4 (4.2%)	
Long course	31 (5.3%)	4 (4.2%)	4 (4.2%)	
Postoperative	2 (0.3%)	1 (1.1%)	0 (0.0%)	
Type of chemotherapy				0.180^c^
None	371 (64.0%)	56 (58.9%)	68 (71.6%)	
Neoadjuvant	37 (6.4%)	5 (5.3%)	3 (3.2%)	
Adjuvant	169 (29.1%)	34 (35.8%)	24 (25.3%)	
Palliative	3 (0.5%)	0	0	
Procedure				0.585^c^
Anterior resection	314 (54.1%)	44 (46.3%)	45 (47.4%)	
Sigmoid colectomy	24 (4.1%)	6 (6.3%)	1 (1.1%)	
Left colectomy	14 (2.4%)	2 (2.1%)	3 (3.2%)	
Right colectomy	149 (25.7%)	34 (35.8%)	34 (35.8%)	
Extended right colectomy	35 (6.0%)	3 (3.2%)	3 (3.2%)	
Hartmann’s procedure	5 (0.9%)	1 (1.1%)	1 (1.1%)	
APR^4^	27 (4.7%)	3 (3.2%)	7 (7.4%)	
Total/subtotal colectomy	5 (0.95%)	1 (1.1%)	1 (1.1%)	
Panproctocolectomy	7 (1.2%)	1 (1.1%)	0 (0.0%)	
Stoma				0.301^c^
No stoma	345 (59.5%)	64 (67.4%)	63 (66.3%)	
Temporary ileostomy	183 (31.6%)	23 (24.2%)	24 (25.3%)	
Permanent ileostomy	9 (1.6%)	2 (2.1%)	0 (0.0%)	
Temporary colostomy	7 (1.2%)	2 (2.1%)	0 (0.0%)	
Permanent colostomy	36 (6.2%)	4 (4.2%)	8 (8.4%)	

^1^Body mass index, ^2^American Society of Anesthetists, ^3^American Joint Committee on Cancer, ^4^abdominoperineal resection

^a^Mann–Whitney *U* test, ^b^Pearson’s chi-square test, ^c^Fisher’s exact test

Supervised laparoscopic training sessions were offered to colorectal consultants and board-certified fellows who already had experience in open or laparoscopic abdominal surgery but had not yet received sufficient formal training to perform laparoscopic colorectal procedures independently. So, the trainees were already colorectal specialists and were competent in performing open cancer resections on their own. Therefore, the focus of the training was on the laparoscopic method and not on basics concerning colorectal tumour surgery. Beginning in 2008, colorectal consultants were taught adhering to the framework established by The National Training Programme in Laparoscopic Colorectal Surgery in the UK. Prior to operating on patients, pre-clinical training consisting of wet-lab or cadaveric courses was a basic requirement so that trainees were familiar with the laparoscopic set-up, handling of tissue, dissection techniques and consecutive steps of a specific procedure [14]. The role of the trainer who was scrubbed-in was to assist the trainee, guiding him and demonstrating solutions in difficult situations as required. A structured stepwise approach was taught, consisting in generic task zones (exposure, dissection of vascular pedicle, mobilisation, anastomosis) and corresponding detailed task steps [15]. These included all aspects of patient safety, positioning, surgical site access, exposition, identifying anatomical key structures, dissecting in the correct embryonic planes, ligation of the vessels at the appropriate level, preservation of the autonomic nerves and performing the anastomosis. After 2008, trainees enrolled in the English National Training Programme were assessed using validated personalised online forms (www.lapco.nhs.uk) to measure their proficiency and progress in order to finally decide on their capability to perform the procedure independently. Inhouse fellows not enrolled in the English National Training Programme (e.g. international fellows) were taught and assessed adhering to the same principles but without online assessment. Each key step was assessed by a score of one to six points. One point given meant that the trainer had to perform the step himself. Six points given meant that the trainer felt he could not have done it better. An in-depth analysis of the development, validation and implementation of these forms is provided by Miskovic et al. [15]. As no validation forms existed in the first 2 years of the study period and international inhouse fellows were not formally enrolled in the National Training Programme, this data was not consistently available and therefore not analysed.

### Statistical methods

Propensity-score matching was performed using PS Match (1:1, near neighbour, no discards, no calliper) by Felix Thoemmes [16]. The matching result was assessed by mean standardised differences between the covariates used for matching, the overall balance test (Hansen and Bowers 2010) and the multivariate imbalance measure L1 (Iacus, King and Porro 2010). Categorical baseline and outcome variables after matching were compared using Pearson’s chi-square or Fisher’s exact test as appropriate. Additionally, odds ratios and confidence intervals were provided where appropriate calculated according to Altman (1991). Quantitative variables were displayed as median and range (minimum-maximum) because normal distribution was not assumed. Therefore, the non-parametric Mann–Whitney *U* test for independent samples was used for statistical comparisons. In contrast to baseline variables, no Bonferroni correction was applied for pre-specified outcome variables. Time-to-event analyses (overall survival and disease-free survival) were analysed using Kaplan–Meier curves and compared using the Log-rank test. Hazards ratios (HR) and 95% confidence intervals (95% CI) were calculated using a univariate Cox proportional hazard model. Time-to-recurrence was analysed using a cumulative incidence plot accounting for death as a competing risk and groups were compared using the Gray test [17]. A competing risk regression (Fine and Gray 1999) calculating the HR and 95% CI was also performed. The median follow-up time was calculated using the reverse Kaplan–Meier method [18]. The null hypothesis, namely that there would be no difference in short- and long-term outcomes between proctored and non-proctored colorectal cancer resections, was to be rejected at a *p*-value ≤ 0.05. As a sensitivity analysis, a confirmatory Cox proportional hazard model adjusting for the matching covariates and other baseline covariates in case of a *p* < 0.25 in univariate analysis was run for the primary outcome overall survival using the full sample size meeting the inclusion criteria (including also the unmatched cases). All analyses were performed in IBM SPSS 20 and R version 2.15.1, mainly using the survival, cmprsk and MatchIt package.

### Sample size considerations

As this is a retrospective analysis and the final sample size (*n* = 95 in each group after matching) was given by the number of supervised training cases, a post hoc power analysis was performed. As the events of interest (e.g. mortality) were rare in both groups and the results were all non-significant, the post hoc power of the study was low (<80%). This is also reflected by large confidence intervals as provided for dichotomous outcome variables (Table 2). Sample sizes of at least 400 cases in each group would be necessary for most dichotomous outcomes to reach a post hoc power of 80% at a significance level of 5%. Although post hoc power analyses will mostly result in low power when there were no significant outcomes, it advises cautious interpretation of the study considering these limitations. Therefore, the primary overall survival outcome of the study has also been analysed using a Cox proportional hazard model where the disadvantages of matching, namely deflating the sample size, were absent. According to Cenzer et al., our sample size would be classified as a moderately small sample and a propensity score matching with one-to-one matching was considered to provide the best matching performance [19]. Therefore, inflating the sample size by one to multiple matching was not favoured.

**Table 2:** Short-term outcomes of matched non-training case and training case colorectal cancer resections

	Non-training cases (*n*=95)	Training cases (*n*=95)	*p*	*OR* (95% *CI*)^c^
Operation time in minutes (median, range)	160 (70–420)	180 (30–360)	0.072^a^	
Blood loss in ml (median, range)	10 (0–500)	10 (0–200)	0.224^a^	
Conversion			0.497^b^	5.40(0.24–107)
No	95 (100%)	93 (97.9%)		
Yes	0 (0%)	2 (2.1%)		
Length of stay in days (median, range)	5 (2–29)	4 (2–30)	0.751^a^	
30-day mortality			0.497^b^	0.33 (0.01–8.2)
No	94 (98.9%)	95 (100%)		
Yes	1 (1.1%)	0 (0%)		
Readmission			0.795^b^	0.76 (0.28–2.10)
No	86 (90.5%)	88 (92.6%)		
Yes	9 (9.5%)	7 (7.4%)		
Complications			0.097^b^	0.66 (0.32–1.38)
No complications (without mortality)	74 (77.9%)	80 (84.2%)		
Minor leak	6 (6.3%)	3 (3.2%)		
Major leak	2 (2.1%)	0 (0.0%)		
Prolonged ileus	3 (3.2%)	1 (1.1%)		
Prolonged pain	4 (4.2%)	0 (0.0%)		
Wound infection	0 (0.0%)	5 (5.3%)		
Abdominal sepsis	1 (1.1%)	1 (1.1%)		
High stoma output	1 (1.1%)	1 (1.1%)		
Stoma fashioning	1 (1.1%)	2 (2.1%)		
Bleeding	1 (1.1%)	1 (1.1%)		
Urinary tract affection	1 (1.1%)	1 (1.1%)		
Medical condition	1 (1.1%)	0 (0.0%)		
Reoperation			1.000^b^	0.49 (0.08–5.54)
No	93 (97.3%)	94 (98.9%)		
Yes	2 (2.1%)	1 (1.1%)		
Clavien–Dindo graded complications			0.068^b^	0.74 (0.38–1.46)
No complications	70 (73.7%)	75 (78.9%)		
1	2 (2.1%)	9 (9.5%)		
2	17 (17.9%)	9 (9.5%)		
3a	2 (2.1%)	1 (1.1%)		
3b	2 (2.1%)	1 (1.1%)		
4a	0 (0.0%)	0 (0.0%)		
4b	1 (1.1%)	0 (0.0%)		
5	1 (1.1%)	0 (0.0%)		
Major complications (≥3b)			0.368^b^	0.29 (0.03–2.21)
No	91 (95.8%)	94 (98.9%)		
Yes	4 (4.2%)	1 (1.1%)		
R stage			0.444^b^	0.39 (0.07–2.05)
R0	90 (94.7%)	93 (97.9%)		
R1 or R2	5 (5.3%)	2 (2.1%)		
Lymph node harvest (median, range)	17 (5–56)	15 (3–48)	0.057^a^	

^a^Mann–Whitney *U* test, ^b^Fisher’s exact test, ^c^odds ratio (OR) with 95% confidence intervals (95% CI) calculated according to Altmann 1991

## Results

From 22.9.2006 to 11.2.2016, a total of 805 laparoscopic colorectal resections for cancer were performed (non-training cases) or used for supervised training sessions (training cases) by a single surgeon (trainer). After exclusion of palliative, urgent and open or robotic cases, a total of 675 resections were performed from 13.10.2006 to 4.2.2016, of which 95 entire procedures were formal training cases for consultants or fellows in training (trainees). These 95 training cases were 1:1 propensity-score matched to non-training cases for age, ASA grade, tumour site (rectum, left-sided colon, right-sided colon) and AJCC tumour stage. None of the matched covariates exhibited an imbalance of the standardised mean difference of greater than 0.25 (│*d*│>0.25), resulting in a good overall balance test (*p* = 0.952, Hansen and Bowers 2010). The multivariate imbalance measure L1 (Iacus, King and Porro 2010) decreased by matching from 0.654 to 0.411, also reflecting a considerable increase in balance by matching. The baseline parameters of the unmatched and the matched groups are displayed in Table 1. None of the baseline characteristics showed significant differences after matching. Comparing non-training cases to training cases, the median age was 72 vs 71 years, female gender accounted for 42.1% vs 51.6% and ASA grade 2 was 69.5% vs 65.3%, whereas in both groups, ASA grade 3 was 21.1% and body mass index (BMI) was 26 kg/m^2^. Rectal tumours were more frequent in the training case group (33.7% vs 29.5%, not significant [n.s.]), whereas the non-training case group contained more AJCC stage 3 tumours (37.9% versus 28.4%, n.s.), explaining the higher proportion of adjuvant chemotherapy there (35.8% versus 25.3%). The distribution of the surgical procedures was well balanced, of which anterior resections and right colectomies were the most commonly performed operations in the non-training case (82.1%) and training case (83.2%) groups.

The secondary outcomes are listed in Table 2. In general, there were no significant differences in secondary outcomes given the limitation of low power due to small sample sizes reflected by large confidence intervals. Two conversions (2.1%) in the training case and none in the non-training case group (n.s.) were observed. Median blood loss was 10 ml in both groups. There was a trend towards longer operation times in the training case group (180 versus 160 min, *p* = 0.072), whereas the length of hospital stay was similar with 5 days in the non-training case and 4 days in the training case group (n.s.). The training case group had fewer readmissions (7 versus 9 cases, n.s.) and complications (15 versus 21 cases, *p* = 0.097; *OR* 0.66 [0.32–1.38]). There was no major leak in the training case compared to two major leaks in the non-training case group. Two of the non-training and one of the training cases had to be taken back to the theatre. When grading the complications according to Clavien–Dindo, including one 30-day mortality case in the non-training case group, the training case group showed a tendency towards fewer and less severe complications (*p* = 0.068). Thus, the training case group had only one severe complication defined as grade 3b or higher compared to four major complications in the non-training case group (*OR* 0.29 [0.03–2.21], *p*=0.37). Major complications in the non-training case group were caused by two major leaks (ASA 2) and a mechanic ileus (ASA 2) in anterior resections which had to be re-operated, as well as a death (ASA 3) on postoperative day 7 in a rectal amputation case due to a medical condition. The single major complication in the training-case group was caused by stoma-related problems in a rectal amputation case (ASA 3) that needed re-fashioning of the stoma (Clavien–Dindo 3b). Lymph node harvest was higher in the non-training case group (17 vs 15, *p* = 0.057).

### Time-to-event analysis (primary outcomes)

**Fig. 1:**
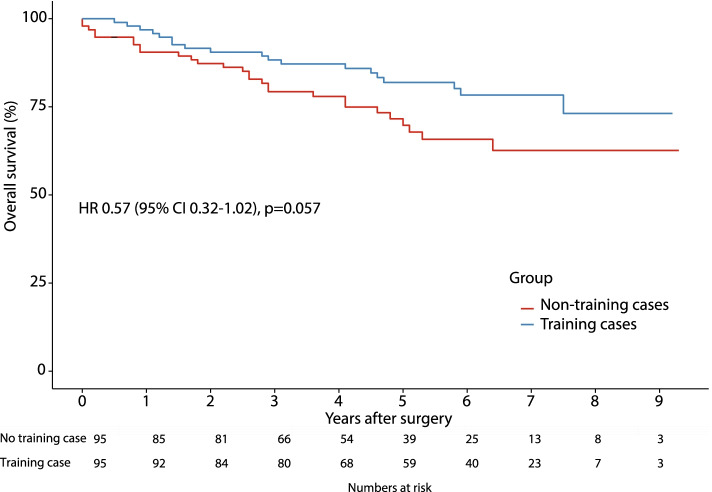
Kaplan-Meier curve of overall survival of training case and non-training case laparoscopic colorectal cancer resections in curative intent. Hazard ratio (HR) and 95% confidence interval (95% CI) were calculated by a univariate Cox proportional hazard model

**Fig. 2:**
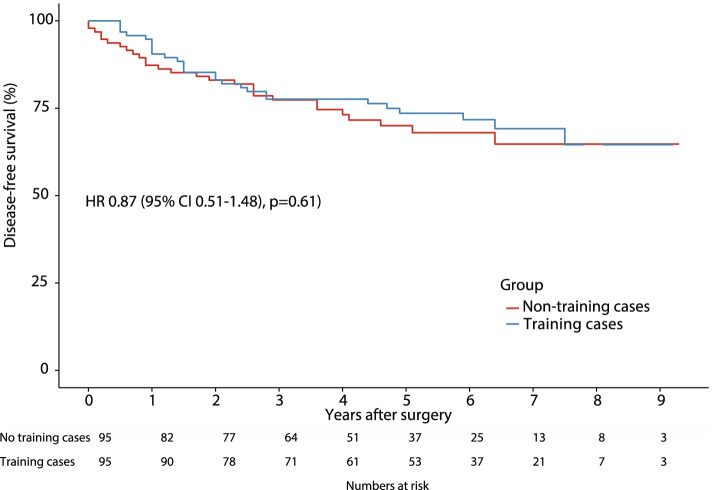
Kaplan-Meier curve of disease-free survival of training case and non-training case laparoscopic colorectal cancer resections in curative intent. Hazard ratio (HR) and 95% confidence interval (95% CI) were calculated by a univariate Cox proportional hazard model

Median follow-up and 95% confidence intervals of the non-training and training case group were 5.3 (4.8–6.1) and 6.0 (5.8–6.5) years, respectively (*p* = 0.276, Log-rank test). During the follow-up time, 11 (11.6%) recurrences in the non-training and 19 (20%) recurrences in the training case group were observed. Although the incidence of local recurrences was the same (one in each group), distant recurrences were more common with 17 (17.9%) in the training case versus only 9 (9.5%) in the non-training case group. Accounting for death as a competing risk for recurrence, the cumulative 5-year incidence of recurrence was 12.5% in the non-training and 19.7% in the training case group (*p* = 0.151, Gray test). The hazard ratio of training cases to have recurrences was 1.72 (95% *CI* 0.822–3.58) compared to a non-training case (*p* = 0.15) when calculated by a competing risk regression (Fine and Gray, 1999). The 5-year overall survival was 71.6% (95% *CI* 62.4–82.2) in the non-training and 81.9% (95% *CI* 74.2–90.4) in the training case group (Log-rank test: *X*^2^(1) = 3.7, *p* = 0.055). The 5-year recurrence-free survival was 70.0% (95% *CI* 60.8–80.6) in the non-training and 73.6% (95% *CI* 64.9–83.3) in the training case group (Log-rank test: *X*^2^(1) = 0.3, *p* = 0.605). The corresponding Kaplan–Meier plots with at-risk tables are shown in Figs. 1 and 2. The hazard ratios using a univariate Cox proportional hazard model were 0.57 (95% *CI* 0.32–1.02, *p* = 0.057) for overall survival and 0.87 (95% *CI* 0.51–1.48, *p* = 0.61) for recurrence-free survival.

In the sensitivity analysis for the primary overall survival outcome, the Cox proportional hazard model of the 675 patients meeting the inclusion criteria (154 events) showed that supervised training was not identified as an independent risk factor for death, with a hazard ratio of 0.779 (95% *CI* 0.479–1.267, *p* = 0.314) when adjusted for age, gender, body mass index, ASA and AJCC tumour stage (9 factor levels). Sample size considerations were considered to be satisfied according to the rule proposed by Green in 1991.

## Discussion

This study shows that supervised training of consultants or fellows in laparoscopic colorectal cancer surgery may not adversely affect short- and long-term outcomes when compared to expert-level surgery and when performed in a structured and standardised setting as provided in the analysed single centre. Comparison of laparoscopic colorectal cancer resections where the same expert surgeon is the primary operating surgeon in one group and the supervising trainer in the other group is scarce in the literature. A similar design was investigated by Renwick et al. where a single consultant surgeon performed 150 colorectal resections and compared the outcomes to those of 344 resections performed by trainees supervised by the same consultant in a standardised training environment [20]. Other than higher intraoperative blood loss, longer operation times and more cardiac as well as respiratory complications in the training group, no other significant short-term outcome differences were registered, and the 2-year overall survival rate was similar. However, different from our study, case selection bias was not addressed and trainees consisted also in colorectal residents (not board certified). The study period was from 1995 to 2002 and no laparoscopic procedures were mentioned. More comparable and in support of our results is the study by De’Ath et al. where also a National Laparoscopic Trainer (Lapco) performed 199 and supervised 101 laparoscopic colorectal resections [21]. Blood loss, lymph node count and the rate of conversion, anastomotic leak, readmission, mortality and 2-year recurrence were similar. However, length of stay, stoma formation rate and operative time were higher in the non-training cases which the authors explained by more complex procedures in the trainer cohort because no adjustment for selection bias was applied. Modular-based training and case selection was considered to be the reason for the favourable outcomes in the training group. We minimised selection bias by propensity-score matching excluding overly complex cases which might explain that length of stay, stoma formation rate and operative time were not higher in our non-training cases.

In our study, the characteristics of the trainees regarding their individual learning curve have not been taken into account. Learning curve analysis and its impact on outcome have been investigated in the past [22]. In an analysis of 1194 patients operated on by 114 surgeons by Charles et al., a case-load-dependent learning effect was shown, and multivariate regression analysis revealed a higher probability of intra- and postoperative complications in low-volume surgeons during their learning curve [23]. However, harming the patient by training cases should not be the case anymore. Ideally, training should be safe and produce outcomes unaffected by a training environment. Our study is not about the learning curve, which generally analyses the change in outcome parameters of consecutively operated patients; rather, it concerns the transfer of specific laparoscopic skills from trainer to trainee where the outcomes of the trainees are not compared to their previous cases but to the performance level of their trainer. By matching similar cases operated on by the trainer himself to supervised training cases, meaningful benchmarking was possible. Meanwhile, it has been demonstrated by Hanna et al. that laparoscopic colorectal surgery can be implemented with continuing good outcomes in the long-term also after completion of supervised training [24]. Because our study predominately contained the early learning curve cases and often the very first laparoscopic colorectal procedure of consultants or fellows in training, it is reassuring to learn in retrospect that not only are the short-term results comparable to the trainer’s standard but also the long-term results. Procedural standardisation by the surgeon qualifying for being a trainer in the first place followed by a standardised and structured supervised clinical training seems to be the correct approach to transfer operational skills without harming the patient.

Although not significant, there was a tendency towards more severe postoperative complications in the non-training group. Whereas in the training group, only a single complication graded ≥3b (Clavien–Dindo classification) occurred, four complications graded ≥3b were noted in the non-training group: two major leaks and a closed loop ileus required revision surgery; one patient (ASA 3) died due to a medical condition without preceding surgical complications. This surprising outcome was not explained by an imbalance in age, ASA grade, body mass index or type of procedure. Although some more rectal resections (32 vs 28) were performed in the training group, more of them were abdomino-perineal resections, thus eliminating the risk of anastomotic leakage. However, the total number of anastomoses (87 anastomoses in each group) was identical in both groups as were the ASA 3 cases (20 in each group) reflecting a good balance by matching. Despite an in-depth review of these severe complication cases in the non-training group, we could not identify risk factors for these three clearly surgical-related complications. In a systematic review and meta-analysis of trainee- versus expert surgeon-performed colorectal resections by Kelly et al. where a total of 8845 colorectal resections were performed by experts and 5499 by trainees, they identified a significant lower leak rate in the cases performed by supervised or unsupervised trainees (odds ratio 0.72 [95% *CI* 0.56–0.92], *p* = 0.010) [25]. However, when they compared only supervised trainees to experts, there was no significant difference anymore (*OR* 0.77 [95% *CI* 0.57–1.03], *p* = 0.080). This is a surprising and unexpected finding as it would suggest that unsupervised trainees have lower anastomotic leak rates. However, once more, most studies included in the meta-analysis did not adjust for selection bias. Furthermore, emergency cases were not excluded and the definition of supervision was heterogenous ranging from undefined to active assistance by the expert. Cases operated on by trainees not needing any kind of supervision tend to be a selection of straightforward cases which might explain the superior results of unsupervised cases. In a standardised and well-planned supervised training, cases are selected and sufficient time is scheduled for the procedure, then the trainee is assisted by the experienced trainer and often by an additional advanced registrar or fellow. Expert camera work and counter-traction optimally exposing the target facilitate the procedure to a significant degree. These advantages are often lacking when the trainer is operating on his own, assisted only by novices. In addition, although our trainees were not yet fully proficient in laparoscopic colorectal surgery, they were operatively skilled colorectal specialists and could very well point out issues the expert trainer might not be aware of. Also, the fashioning of an anastomosis in anterior resections where the two major leaks occurred in our non-training group is quite similar to open surgery. Some more T4 tumours, although only one case was T4b, were resected in the non-training group (13 versus 4), potentially further increasing the complexity of the surgery and the potential for complications. Although the higher number of complications in the non-training case group is not fully explained and needs further monitoring, one might agree that training cases did not increase complications in our setting.

On average, the operation time was shorter by 20 min in the non-training group (*p* = 0.072), a result that most probably is explained by the different levels of expertise. However, as mentioned above, De’Ath et al. found longer operating times in the non-training cases which were attributed to more complex cases in this group [21]. Careful case selection for training is common practice and reasonable in order not to demotivate the trainee. The only case leading to major complications in our training group was a case with previous surgery where conversion was necessary, obviously not an ideal training case but reflecting reality. We did not match the groups accounting for previous surgery as the kind of previous surgery was not specified in our database and the impact of previous surgery is often unpredictable unless extensive adhesions have been documented previously. The fact, that operation times tended to be shorter in our non-training case group as one would expect, may indicate that matching indeed reduced selection bias and eliminated overly complex cases.

The overall survival tended to be better in the training case group, although not reaching significance. On the other hand, more recurrences occurred in the training group, which appears to be a contradictory result. More T4 tumours and R1 resections, as well as N2 stages, were observed in the non-training case group. Therefore, more patients received adjuvant chemotherapy in this group. Effective chemotherapy and perhaps a stage migration effect may partially explain the longer time to recurrence in the non-training group. Due to the higher lymph node count in non-training cases, more positive nodes might have been detected, leading to an upstaging and consecutive administration of adjuvant chemotherapy. This could also explain the higher number of distant metastases in the training group, which had received less adjuvant treatment in general. Endo et al. confirmed in a multivariate analysis of left-sided colon tumours that an adequate lymph node harvest (≥12) and adjuvant chemotherapy were independent prognostic factors for survival [26]. In the above mentioned meta-analysis by Kelly et al., they could not find cancer-specific survival differences between trainees (supervised and unsupervised) and experts [25]. However, the meta-analysis showed significantly less R0 resections in the supervised training case collective which is in contrast to our findings. Recently, overall survival prognostic models including blood cholesterol-to-lymphocyte ratios, carcinoembryonic antigen levels or RNA-binding proteins have been investigated and may help to further delineate the individual risk profile in the future [27–29]. None of these newer and often still experimental risk factors was consistently investigated in our study group. Therefore, no further risk stratification using those parameters concerning overall survival was possible. However, without any doubt, emerging molecular markers and risk factors certainly continue to allow a more tailored analysis and treatment in the future. In sum, slight imbalances in the groups may explain the non-significant differences in our outcomes as supervised training was also not an independent risk factor for death in the Cox proportional hazard model of the unmatched cohort of 675 samples in the sensitivity analysis.

Currently, laparoscopic surgery has been successfully implemented in most colorectal services, and the requirement for laparoscopic training of surgeons primarily experienced in open colorectal surgery is decreasing. For many younger surgeons, laparoscopy has been the default method from the very beginning of their training and open procedures are the exception. Knowing that structured and standardised training pays off in the long term, the focus should now be on the ongoing implementation of newer techniques, which, if demonstrated to be valid and better, should be made accessible to a broad range of surgeons. Techniques like transanal total mesorectal excision (TME) or robotic colorectal resections still incorporate many principles of conventional laparoscopy and skill translation to another minimally invasive technique seems to be even more straightforward than from open to laparoscopic surgery. This has been demonstrated by the authors in the case of robotic total mesorectal excision (TME) where only 15 training cases were necessary to achieve the same expert level as in conventional laparoscopic TME [30]. Because modern non-surgical treatment concepts like neoadjuvant immunotherapy are evolving with promising results [31], the role of surgery has to be redefined constantly. However, up to now, surgery is still an important treatment modality in most colorectal cancer patients and teaching laparoscopic cancer surgery in all parts of the world remains of interest.

This study has limitations due to its retrospective nature and low sample size as well as power. It might be debated that there could be an overall survival benefit in favour of the training group if more cases had been included. The sensitivity analysis by multivariable regression using the full sample, however, could not confirm this assumption. Further monitoring and investigations of training-related outcomes using larger samples will be needed.

## Conclusions

Supervised training in laparoscopic colorectal cancer resections of colorectal specialist surgeons or fellows by an expert trainer seems to be safe and may not adversely impact the short- and long-term outcomes of selected training cases and when adhering to a standardised training framework.

## Data Availability

The datasets used and/or analysed during the current study are available from the corresponding author on reasonable request.

## Abbreviations

AJCC: American Joint Committee on Cancer
ASA: American Society of Anesthetists
BMI: Body mass index
Lapco: National Training Programme for Laparoscopic Colorectal Surgery
